# Multimodality annotated hepatocellular carcinoma data set including pre- and post-TACE with imaging segmentation

**DOI:** 10.1038/s41597-023-01928-3

**Published:** 2023-01-18

**Authors:** Ahmed W. Moawad, Ali Morshid, Ahmed M. Khalaf, Mohab M. Elmohr, John D. Hazle, David Fuentes, Mohamed Badawy, Ahmed O. Kaseb, Manal Hassan, Armeen Mahvash, Janio Szklaruk, Aliyya Qayyum, Abdelrahman Abusaif, William C. Bennett, Tracy S. Nolan, Brittney Camp, Khaled M. Elsayes

**Affiliations:** 1 Departments of Imaging Physics, The University of Texas MD Anderson Cancer Center, Houston, TX 77030 USA; 2 Department of radiology, Mercy catholic medical center, Darby, PA 19023 USA; 3 Departments of Gastrointestinal Oncology, The University of Texas MD Anderson Cancer Center, Houston, TX 77030 USA; 4 Departments of Interventional Radiology, The University of Texas MD Anderson Cancer Center, Houston, TX 77030 USA; 5 Departments of Body Imaging, The University of Texas MD Anderson Cancer Center, Houston, TX 77030 USA; 6 Department of Biomedical Informatics, University of Arkansas for Medical Sciences, Little Rock, AR 72205 USA; 7 Department of radiology, Baylor college of medicine, TX 77030 Houston, USA

**Keywords:** Liver cancer, Medical imaging, Oncogenesis

## Abstract

Hepatocellular carcinoma (HCC) is the most common primary liver neoplasm, and its incidence has doubled over the past two decades owing to increasing risk factors. Despite surveillance, most HCC cases are diagnosed at advanced stages and can only be treated using transarterial chemo-embolization (TACE) or systemic therapy. TACE failure may occur with incidence reaching up to 60% of cases, leaving patients with a financial and emotional burden. Radiomics has emerged as a new tool capable of predicting tumor response to TACE from pre-procedural computed tomography (CT) studies. This data report defines the HCC-TACE data collection of confirmed HCC patients who underwent TACE and have pre- and post-procedure CT imaging studies and available treatment outcomes (time-to-progression and overall survival). Clinically curated segmentation of pre-procedural CT studies was done for the purpose of algorithm training for prediction and automatic liver tumor segmentation.

## Abstract (summary)

**Table:** 

Measurement(s)	Image Segmentation • hepatocellular carcinoma
Technology Type(s)	Neural Network • Multiphasic Computed Tomography of the Abdomen
Sample Characteristic - Organism	multiphasic CT of the abdomen
Sample Characteristic - Location	contiguous United States of America

## Background & Summary

Hepatocellular carcinoma (HCC) is the most common primary liver neoplasm, with an incidence of 42,810 newly diagnosed cases in the United States in 2020^1^. The rates of HCC have doubled over the past two decades and are anticipated to continue to increase owing to increasing risk factors such as liver cirrhosis, steatohepatitis, and obesity^2–4^. Despite close HCC surveillance, 70%–80% of HCC cases are diagnosed at advanced stages when they are unresectable. Treatment of unresectable HCC includes transarterial chemo-embolization (TACE) and systemic sorafenib therapy^5–7^.

TACE selectively delivers chemotherapy to targeted liver tumors, taking advantage of the fact that HCC primarily receives its blood supply from the hepatic artery, while the liver parenchyma primarily receives its blood supply from the portal vein. This technique spares the healthy liver parenchyma from being damaged by the chemotherapy^5^. The Barcelona Clinic Liver Cancer (BCLC) staging system is recommended for HCC patient stratification and treatment selection. It includes patient performance status, severity of the underlying liver disease using clinical and laboratory markers of liver synthetic performance (Child Pugh Grading [CPG]), and extent of tumor including its size, number of tumor foci, metastasis, and vascular invasion^7,8^. Candidates for TACE therapy include BCLC stage B patients (with intermediate HCC) and patients waiting for a liver transplant who underwent TACE as bridging therapy. The median survival duration of patients with HCC is 16 months from diagnosis for intermediate cases, which decreases to 6–8 months for advanced cases^8–10^.

TACE is an invasive procedure with several potential adverse effects, including treatment failure, organ failure, and even death. In addition, studies have shown that up to 60% of patients with HCC who undergo TACE do not benefit from the procedure in spite of multiple sessions, causing financial and emotional burdens for the patient^11–13^. Clinical models are being developed for the prediction of TACE outcomes and the selection of patients who will benefit from the procedure. However, the high variability in TACE response has encouraged researchers to develop complicated predictive models using histological tumor markers like vascular endothelial growth factor, biological markers like alpha-fetoprotein, or a combination of both^13–17^. However larger patient sample sizes are needed to validate their prognostic value. In addition, poor detection rate of alpha-feto protein in patients with small residual size tumor after TACE may limit its prognostic significance^1,18^. Artificial intelligence (AI) and its sub-classes, deep learning, machine learning, and radiomics, have been used in imaging for various tasks including classification, segmentation, and detection. Prediction is an important task that has been shown to be best solved by AI. This includes prediction of disease severity, prediction of treatment response, and prediction of disease progression^19–22^. AI-based prediction models are usually not included in reports from large multi-center randomized trials, limiting the development of widely used predictive models. Furthermore, most of the in-house-developed models lack generalizability to other institutions or even with different datasets. Therefore, external validation of AI models is an integral step for the model to be widely used in day-to-day clinical practice^23–26^. Although large imaging datasets have been made available to the public to be used in further research steps or in re-generating the original results, treatment-related datasets are scarce owing to the complexity of various treatments and the variability in tumor grading and treatment selection. Recent advances in patient de-identification and image registration have allowed for the creation of different imaging databases within The Cancer Imaging Archive (TCIA) that may offer a better opportunity for treatment response validation^27,28^.

Here, we present the HCC-TACE collection, a single-institution collection of 105 patients with confirmed HCC treated at The University of Texas MD Anderson Cancer Center from 2002 to 2012. The HCC-TACE collection integrates de-identified, comprehensive clinical data with diagnostic imaging and its manual segmentation and makes these data publicly available to researchers. Unique to this dataset is the inclusion of TACE procedure details, imaging before and after the procedure, manual segmentation (liver parenchyma, viable and necrotic tumor tissue, intra-hepatic vessels, and aorta), radiological measures of treatment response by experts in abdominal imaging, as well as patient outcomes after the procedure in the form of overall survival (OS) and time-to-progression (TTP). These data were used in prior publications, which evaluated prediction of TACE procedure outcomes using pre-procedural imaging and proposed convolutional neural network architecture for segmentation of HCC and automation of the prediction process^29,30^. The open access to this data allows for inter-institutional comparisons of non-randomized patient, treatment, and outcome data, in addition to the development of new architectures and models with higher performance and the external validation of other developed models using our patient cohort.

## Methods

### Study cohort and patient selection

To develop this dataset, the MD Anderson Cancer Center institutional database was searched for patients with HCC treated from November 2002 to June 2012. Inclusion criteria were patients with HCC who (1) underwent TACE as the sole first-line therapy or initial bridging therapy, (2) had available multi-phasic contrast-enhanced CT images with liver protocol obtained prior to TACE (Pre-procedural scans), (3) had available multi-phasic contrast-enhanced CT images with liver protocol obtained within 14 weeks from the TACE procedure (post-procedural scans), and (4) had CT images of acceptable quality with no obvious artifacts. Patients undergoing TACE who had more than one HCC focus were excluded, so that calculation of OS and TTP depends only on one tumor and with no confounders. This study was approved by our institutional review board; written informed consent was waived due to the retrospective nature of the study. Pre-procedural CT images were obtained 1–12 weeks prior to the first TACE session (average 3 weeks).

The final patient cohort identified in our institutional database (N = 105) was 68 male patients (average age, 66.4 years [range, 31–88 years]) and 37 female patients (average age, 69.6 years [range, 46–93 years]). Risk factors for development of HCC were reported for each patient, including hepatitis, smoking, alcohol use, diabetes, cirrhosis, and family history of either liver disease or cancer. Multiple grading systems were recorded for our cohort to enhance the usage of our dataset: CLIP score^31^, Okuda score^32^, TNM staging system^33^, and BCLC staging system^34^. In addition, performance status according to the Eastern Cooperative Oncology Group (ECOG) scale, CPG, alpha-fetoprotein level, and tumor extent (tumor size, vascular and lymph node invasion, distant metastasis) were reported.

### Image acquisition

All patients underwent contrast-enhanced CT of the abdomen, with liver protocol on 16–, or 64–detector row CT scanners (LightSpeed; GE Healthcare, Waukesha, WI, USA). A pre-contrast scan was obtained, followed by an arterial phase scan 17 seconds after peak enhancement (using bolus tracking) of the aorta after injection of contrast medium. The porto-venous phase was scanned at 60 seconds. Images were acquired with the following scanner parameters: CT tube voltage of 120–140 KVp; Tube current of 150–630 mA; slice thickness of 0.63–5 mm; Pitch of 0.9–0.98; revolution time of 0.40–0.80 seconds; table speed of 18.75–39.38 mm/gantry rotation and field of view of 360–460 mm. The injection rate of contrast medium was 3–5 ml/sec. Standard image reconstruction algorithm was used in all cases. A total of 621 CT series (pre-procedural and post-procedural multi-phasic scans) from 105 patients were examined.

### Assessment of tumor response

Tumor response to TACE was assessed using European Association for the Study of the Liver (EASL), Response Evaluation Criteria in Solid Tumors (RECIST) 1.1, and modified RECIST (mRECIST) guidelines. All pre-and post-procedural studies were reviewed by three different board-certified radiologists (K.M.E. [reader 1], J.S. [reader 2], and A.Q. [reader 3]), each with more than 20 years of experience in abdominal imaging. They independently measured tumors in both pre- and post-procedural studies, taking into consideration tumor viability and enhancement in the arterial phase. EASL measurements were recorded by reader 1 only, RECIST 1.1 and mRECIST measurements were recorded by all the three readers.

### TACE procedure and study endpoints

Patients undergoing TACE were administered one of the following chemotherapy regimens: (a) doxorubicin in 20- to 100-mg drug-eluting beads (33 lesions; LC Beads, DEBDOX, BTG International, London, England) or (b) cisplatin, doxorubicin, and mitomycin C (100, 50, and 10 mg, respectively; 55 lesions). Details about the TACE procedure were missing in 17 patients. The patient cohort was monitored longitudinally with post-procedural CT. Each lesion was monitored for progression based on radiology reports. The TTP was defined as the number of weeks from the treatment (TACE) to the date of radiological evidence of progression according to mRECIST criteria. Lesions were considered as censored if (a) there was no progression by the study date, (b) the patient was lost to follow-up, either due to not appearing or died in the meantime, (c) the date of death was before the date of tumor progression, or (d) treatment was changed to something other than TACE. In our previous studies, we divided patients into TACE-susceptible and TACE-refractory groups with a cutoff TTP of 14 weeks. TACE-susceptible patients are those who do not show radiological progression at follow-up CT, while TACE-refractory patients show radiological progression of the tumor.

### Data processing and curation

The AI approach for building a neural network or machine learning model is to extract imaging features from sub-volumes in the study. Segmentation of CT studies has been the main obstacle in creating such models from publicly available datasets as acquiring well-curated data is usually a time-consuming and labor-intensive process that requires dedicated personnel. In order to build our model, segmentation of the tumor (both viable and necrotic volumes) and background liver was done. The porto-venous phase of pre-procedural CT studies was used to simplify lesion assessment, and CT studies were exported in DICOM format and subsequently converted into Neuroimaging Informatics Technology Initiative (NIFTI) format to preserve orientation information and pixel spacing, with simpler headers than standard DICOM format. For each patient study, pre-contrast, arterial, and port-venous images of the pre-procedural scans were registered and re-sampled to the port-venous phase images. Segmentation of intra-hepatic vessels and the abdominal aorta was done as well. Manual segmentation was done using semi-automated segmentation tools available in AMIRA software (FEI, Thermo Fisher Scientific, Hillsboro, OR, USA) by three different radiology residents (A.M., A.M.K., and M.E.) and reviewed by a body imaging radiologist with 20 years of experience (K.M.E.).

**Fig. 1:**
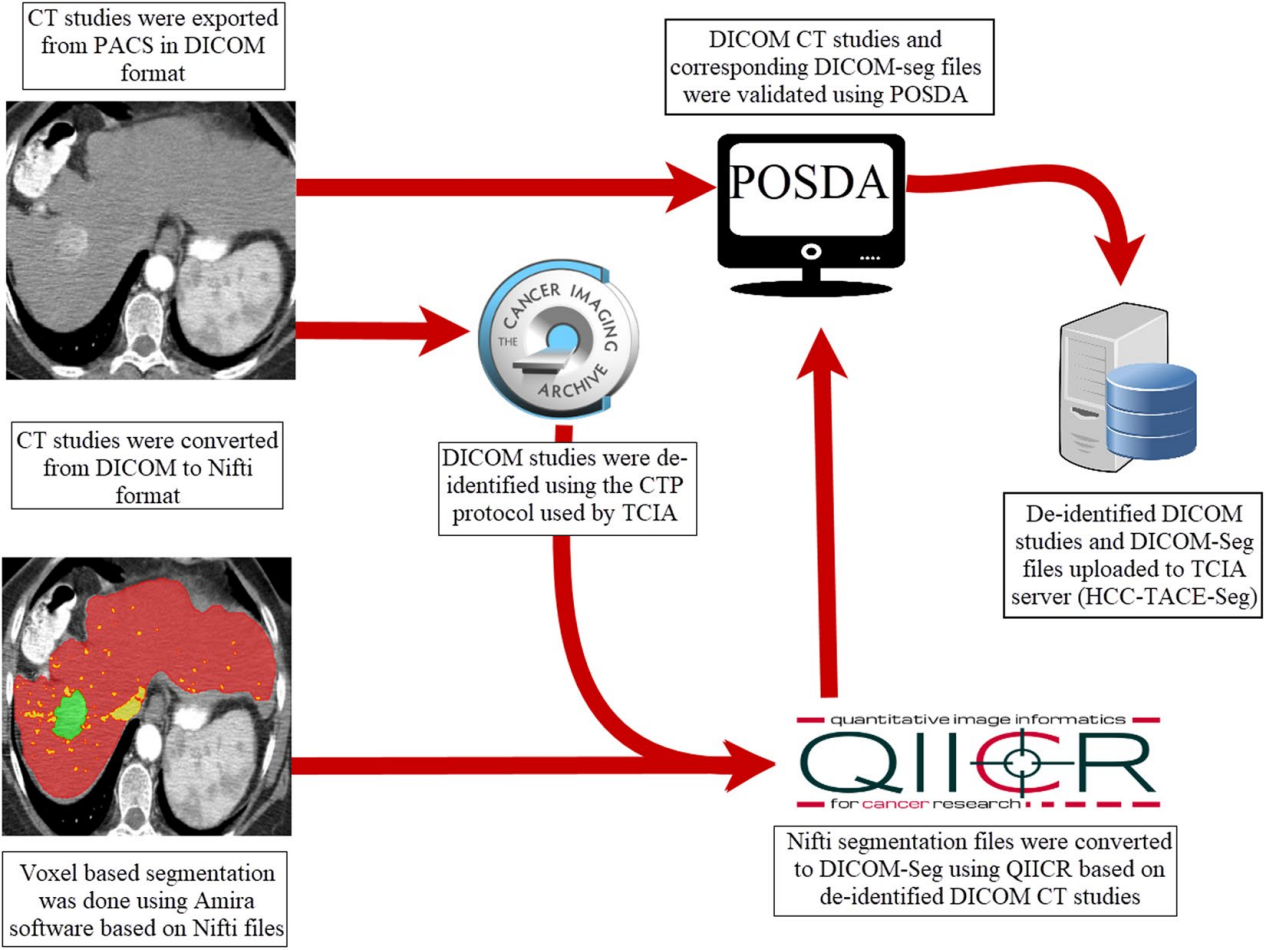
A schematic overview of the process of the dataset curation and processing in TCIA.

The three different segmentations were validated and combined together to produce a single image using the STAPLE algorithm to produce the ground truth segmentation. The STAPLE algorithm uses the different segmentations as an input and generate a binary image of each voxel being the “true” segmentation. This process is achieved on each label^35^. Image registration was done using affine transformation and linear interpolation. All images manipulation, and STAPLE image production were performed using the Convert3D medical image processing tool available with the ITK-SNAP software package^36^. To enhance the generalizability of the dataset, segmented NIFTI files have been converted to DICOM-SEG using the DICOM for Quantitative Imaging (dcmqi) library for Quantitative Image Informatics for Cancer Research (QIICR) in the 3D-Slicer software package^37,38^. The process of data curation and processing is demonstrated in Fig. 1.

### Neural network architecture and training

Our original work^29^ was to automate the treatment response prediction process by automating segmentation of the liver and both viable and necrotic tumor. Two back-to-back convolutional neural networks (CNNs) were constructed for that purpose. The first CNN (CNN1) was built to segment liver tissue from the background using axial images of port-venous phase CT scans; this network was trained on the axial CT images and corresponding liver segmentations from the Medical Image Computing and Computer Assisted Intervention Society Liver Tumor Segmentation (or LiTS) challenge (130 manually labeled CT images; publicly available)^39^. CNN2 was built to segment HCC from the output of CNN1, and it was trained separately on the manual segmentation of our cohort (105 manually labeled images; publicly available). Both CNNs follow the U-Net architecture^40,41^. The output of CNN1 is a CT image with binary classification of the liver tissue; this image serves as input to CNN2, which segments the tumor from the liver mask. Each convolution operation uses a 3 × 3 kernel size and is followed by batch normalization. The rectified linear unit activation function was also used. A dropout (P = 0.5) was used before each convolution in the up-sampling limb of the U-Net. Our CNN architecture and codes can be found in our GitHub repository (https://github.com/fuentesdt/livermask).

## Data Records

Our dataset consists of (a) 51,832 DICOM files from 621 series and 210 studies collected from 105 patients; (b) 105 DICOM-SEG files, each containing segmentation of the liver, tumor (viable and necrotic), intra-hepatic vessels, and aorta of pre-procedural CT images; and (c) a single spreadsheet file including all of the demographic, clinical, and diagnostic data, as well as EASL, RECIST, and mRECIST readings of each pre- and post-procedural CT images.

There are total of 203 series of pre-contrast phase, 204 series of arterial phase, 210 series of porto-venous phase, and 4 series of delayed phase. Of note, there are 48 series that have combined phases in one series owing to technical errors export of DICOM files from our PACS system to a separate research folder for de-identification. Detailed description for each series is found in supplementary table 1. Available corresponding clinical and survival information for the patients is found in tabular format. Table 1 shows selected headers from the spreadsheet and their relevant descriptions. All of the CT Images (stored as de-identified DICOM files), segmentations (stored as DICOM-Seg files), and spreadsheets containing relevant clinical and radiological information are available at TCIA database in this reference^42^.

**Table 1:** Selected headers from the spreadsheet.

Clinical/survival variable	Description
**Interval_BL**	Number of ***Days*** between HCC diagnosis (either by previous imaging or biopsy) and pre-procedural (baseline) CT
**Interval_FU**	Number of ***Weeks*** between follow-up and baseline studies
**TTP**	Number of ***weeks*** between the procedure and the first evidence of tumor progression.
**Death_1_StillAlive orLostToFU_0**	Binary value (0 or 1), “1” denotes patient died at follow-up date, while “0” denotes patient still alive or lost to follow-up
**Age**	Patient age at diagnosis (*not at baseline CT)*
**Pathology**	Degree of differentiation of HCC according to histopathological assessment
**PS_bclc**	Performance status as component for BCLC classification
**CPG**	Child Pugh grading
**Tr_Size**	Actual pathological tumor size in cm
**T_involvment**	Liver involvement by the tumor, either less than 50% involvement of liver or more.
**AFP**	Alpha fetoprotein level (ng/ml)
**CLIP_Score**	CLIP score
**Okuda**	Okuda score
**TNM**	TNM staging
**BCLC**	Barcelona Clinic Liver Cancer classification
**Chemotherapy**	Chemotherapy regimen used for TACE procedure, either DEB-TACE or conventional TACE.

Abbreviations: HCC, hepatocellular carcinoma; CT, computed tomography; BCLC, Barcelona Clinic Liver Cancer; AFP, alpha feto-protein; TACE, transarterial chemo-embolization; DEB-TACE, drug-eluting beads TACE.

## Technical Validation

Patient’s DICOM files were de-identified using curated clinical trial protocol (CTP) developed by medical imaging resource center (MIRC) recommended by radiological society of North America. The program removes all protected health information (PHI) from all DICOM files metadata. It also replaces study, series and image unique identifiers (UIDs) with hashed version so we ensure complete de-identification of the images in accordance with the Health Insurance Portability and Accountability Act (HIPAA)^28^.

The POSDA Tools used by TCIA for technical validation of this DICOM collection. These tools are openly available, and contributions from the research community are encouraged: https://github.com/UAMS-DBMI/PosdaTools. For further detail on the curation of DICOM CT and SEG files using POSDA Tools within TCIA, please refer to https://posda.com.

To support validation of this collection, the capabilities of POSDA Tools were extended in the following way: extract each 3D-slicewise segmentation from a DICOM-SEG file, convert it to a set of 2D contours, and display those contours superimposed on the referenced CT slice for a (non-radiologist) curator’s eye to confirm reasonable alignment and file linkage. Every CT file and CT file referenced by Unique Identifier in the SEG files was confirmed to exist and checked to ensure data completeness; this capability was added in response to a missing CT file error, which affects one segmentation series for Patient ID HCC_001. This missing file corresponds to image slice far from liver, tumor and their masks, so clinical interpretation should be unaffected by this discrepancy.

## Usage Notes

Multiple open-source software can be used to visualize the DICOM-Seg files; we highly recommend using the latest stable version of 3D-Slicer for data visualization after installing “quantitative reporting” extension. Step-by-step guidance for installation and guidance can be found in: https://qiicr.gitbook.io/quantitativereporting-guide/. For the full list of the available software, please visit dcmqi documentation for instructions at: https://dicom4qi.readthedocs.io/en/latest/results/seg/.

## Supplementary information

Detailed description of each CT series uploaded to TCIA

## Data Availability

The POSDA Tools used by TCIA for technical validation of this DICOM collection, are openly available, and contributions from the research community are encouraged: https://wiki.cancerimagingarchive.net/display/Public/Submission+and+De-identification+Overview For further detail on the curation of DICOM CT and SEG files using Posda Tools within TCIA, please refer to https://posda.com. CNN architecture and codes used in this manuscript can be found in our GitHub repository (https://github.com/fuentesdt/livermask).

## References

[1] Armstrong SA, He AR (2020). Immuno-oncology for Hepatocellular Carcinoma: The Present and the Future. Clinics in Liver Disease.

[2] Aly A, Ronnebaum S, Patel D, Doleh Y, Benavente F (2020). Epidemiologic, humanistic and economic burden of hepatocellular carcinoma in the USA: a systematic literature review. Hepatic Oncology.

[3] Kim H-s, El-Serag HB (2019). The Epidemiology of Hepatocellular Carcinoma in the USA. Current Gastroenterology Reports.

[4] Moawad AW (2020). Angiogenesis in Hepatocellular Carcinoma; Pathophysiology, Targeted Therapy, and Role of Imaging. J Hepatocell Carcinoma.

[5] Pesapane F, Nezami N, Patella F, Geschwind J (2017). New concepts in embolotherapy of HCC. Medical Oncology.

[6] Xiang X (2017). Distribution of tumor stage and initial treatment modality in patients with primary hepatocellular carcinoma. Clinical and Translational Oncology.

[7] Marrero JA (2018). Diagnosis, staging, and management of hepatocellular carcinoma: 2018 practice guidance by the American Association for the Study of Liver Diseases. Hepatology.

[8] Liver EAFTSOT (2018). EASL clinical practice guidelines: management of hepatocellular carcinoma. Journal of hepatology.

[9] Singal AG, Pillai A, Tiro J (2014). Early detection, curative treatment, and survival rates for hepatocellular carcinoma surveillance in patients with cirrhosis: a meta-analysis. PLoS Med.

[10] Heimbach JK (2018). AASLD guidelines for the treatment of hepatocellular carcinoma. Hepatology.

[11] Llovet JM (2002). Arterial embolisation or chemoembolisation versus symptomatic treatment in patients with unresectable hepatocellular carcinoma: a randomised controlled trial. The Lancet.

[12] Garwood ER, Fidelman N, Hoch SE, Kerlan RK, Yao FY (2013). Morbidity and mortality following transarterial liver chemoembolization in patients with hepatocellular carcinoma and synthetic hepatic dysfunction. Liver Transplantation.

[13] Sciarra A (2015). TRIP: a pathological score for transarterial chemoembolization resistance individualized prediction in hepatocellular carcinoma. Liver international.

[14] Huang G-W, Yang L-Y, Lu W-Q (2005). Expression of hypoxia-inducible factor 1α and vascular endothelial growth factor in hepatocellular carcinoma: impact on neovascularization and survival. World journal of gastroenterology: WJG.

[15] Yu SJ (2017). Targeted proteomics predicts a sustained complete-response after transarterial chemoembolization and clinical outcomes in patients with hepatocellular carcinoma: a prospective cohort study. Journal of proteome research.

[16] Kim BK (2016). Risk prediction for patients with hepatocellular carcinoma undergoing chemoembolization: development of a prediction model. Liver International.

[17] Jeong SO (2017). Predictive factors for complete response and recurrence after transarterial chemoembolization in hepatocellular carcinoma. Gut and liver.

[18] Guo J-h, Zhu X, Li X-t, Yang R-j (2012). Impact of serum vascular endothelial growth factor on prognosis in patients with unresectable hepatocellular carcinoma after transarterial chemoembolization. Chinese journal of cancer research.

[19] Zhu HB (2021). Deep learning-assisted magnetic resonance imaging prediction of tumor response to chemotherapy in patients with colorectal liver metastases. Int J Cancer.

[20] Wang CJ (2019). Deep learning for liver tumor diagnosis part II: convolutional neural network interpretation using radiologic imaging features. Eur Radiol.

[21] Shui L (2020). The Era of Radiogenomics in Precision Medicine: An Emerging Approach to Support Diagnosis, Treatment Decisions, and Prognostication in Oncology. Front Oncol.

[22] Jin J (2021). Deep learning radiomics model accurately predicts hepatocellular carcinoma occurrence in chronic hepatitis B patients: a five-year follow-up. Am J Cancer Res.

[23] Liu D (2020). Accurate prediction of responses to transarterial chemoembolization for patients with hepatocellular carcinoma by using artificial intelligence in contrast-enhanced ultrasound. European Radiology.

[24] Shaish H (2020). Radiomics of MRI for pretreatment prediction of pathologic complete response, tumor regression grade, and neoadjuvant rectal score in patients with locally advanced rectal cancer undergoing neoadjuvant chemoradiation: an international multicenter study. European Radiology.

[25] Trebeschi S (2019). Predicting response to cancer immunotherapy using noninvasive radiomic biomarkers. Annals of Oncology.

[26] Bi WL (2019). Artificial intelligence in cancer imaging: Clinical challenges and applications. CA: A Cancer Journal for Clinicians.

[27] Clark K (2013). The Cancer Imaging Archive (TCIA): maintaining and operating a public information repository. Journal of digital imaging.

[28] Moore SM (2015). De-identification of medical images with retention of scientific research value. Radiographics.

[29] Morshid A (2019). A machine learning model to predict hepatocellular carcinoma response to transcatheter arterial chemoembolization. Radiology: Artificial Intelligence.

[30] Khalaf A (2019). Hepatocellular carcinoma response to transcatheter arterial chemoembolisation using automatically generated pre-therapeutic tumour volumes by a random forest-based segmentation protocol. Clinical radiology.

[31] Investigators, C. o. t. L. I. P. (1998). A new prognostic system for hepatocellular carcinoma: a retrospective study of 435 patients. Hepatology.

[32] Okuda K (1985). Natural history of hepatocellular carcinoma and prognosis in relation to treatment study of 850 patients. Cancer.

[33] Minagawa M, Ikai I, Matsuyama Y, Yamaoka Y, Makuuchi M (2007). Staging of hepatocellular carcinoma: assessment of the Japanese TNM and AJCC/UICC TNM systems in a cohort of 13,772 patients in Japan. Annals of surgery.

[34] Llovet, J. M., Brú, C. & Bruix, J. in *Seminars in liver disease*. 329–338 (© 1999 by Thieme Medical Publishers, Inc.). 10.1055/s-2007-1007122 10518312

[35] Warfield SK, Zou KH, Wells WM (2004). Simultaneous truth and performance level estimation (STAPLE): an algorithm for the validation of image segmentation. IEEE Trans Med Imaging.

[36] Yushkevich PA (2006). User-guided 3D active contour segmentation of anatomical structures: Significantly improved efficiency and reliability. NeuroImage.

[37] Fedorov A (2016). DICOM for quantitative imaging biomarker development: a standards based approach to sharing clinical data and structured PET/CT analysis results in head and neck cancer research. PeerJ.

[38] Herz C (2017). DCMQI: an open source library for standardized communication of quantitative image analysis results using DICOM. Cancer research.

[39] Bilic, P. *et al*. The liver tumor segmentation benchmark (lits). *arXiv preprint arXiv:1901.04056* (2019).

[40] Chlebus G (2018). Automatic liver tumor segmentation in CT with fully convolutional neural networks and object-based postprocessing. Scientific reports.

[41] Vorontsov E (2019). Deep learning for automated segmentation of liver lesions at CT in patients with colorectal cancer liver metastases. Radiology: Artificial Intelligence.

[42] Moawad AW (2021). The Cancer Imaging Archive.

